# Oxide-Oxide Thermocompression Direct Bonding Technologies with Capillary Self-Assembly for Multichip-to-Wafer Heterogeneous 3D System Integration

**DOI:** 10.3390/mi7100184

**Published:** 2016-10-10

**Authors:** Takafumi Fukushima, Hideto Hashiguchi, Hiroshi Yonekura, Hisashi Kino, Mariappan Murugesan, Ji-Chel Bea, Kang-Wook Lee, Tetsu Tanaka, Mitsumasa Koyanagi

**Affiliations:** 1 Department of Mechanical Systems Engineering, Graduate School of Engineering, Tohoku University, 6-6-12 Aza-Aoba, Aramaki, Aoba-ku, Sendai 980-8579, Miyagi, Japan; hashiguchi@lbc.mech.tohoku.ac.jp (H.H.); ttanaka@lbc.mech.tohoku.ac.jp (T.T.); 2 New Industry Creation Hatchery Center (NICHe), Tohoku University, Sendai 980-8579, Miyagi, Japan; yonekura@bmi.niche.tohoku.ac.jp (H.Y.); murugesh@bmi.niche.tohoku.ac.jp (M.M.); beatrix@bmi.niche.tohoku.ac.jp (J.-C.B.); kriss@bmi.niche.tohoku.ac.jp (K.-W.L.); koyanagi@bmi.niche.tohoku.ac.jp (M.K.); 3 Tohoku-MicroTec Co., Ltd., 6-6-12 Aza-Aoba, Aramaki, Aoba-ku, Sendai 980-8579, Japan; 4 Frontier Research Institute for Interdisciplinary Sciences, Tohoku University, Sendai 980-8579, Miyagi, Japan; kino@lbc.mech.tohoku.ac.jp; 5 Department of Biomedical Engineering, Graduate School of Biomedical Engineering, Tohoku University, Sendai 980-8579, Miyagi, Japan

**Keywords:** multichip-to-wafer, direct bonding, capillary self-assembly, liquid surface tension, 3D integration, CMP, heterogeneous integration

## Abstract

Plasma- and water-assisted oxide-oxide thermocompression direct bonding for a self-assembly based multichip-to-wafer (MCtW) 3D integration approach was demonstrated. The bonding yields and bonding strengths of the self-assembled chips obtained by the MCtW direct bonding technology were evaluated. In this study, chemical mechanical polish (CMP)-treated oxide formed by plasma-enhanced chemical vapor deposition (PE-CVD) as a MCtW bonding interface was mainly employed, and in addition, wafer-to-wafer thermocompression direct bonding was also used for comparison. N_2_ or Ar plasmas were utilized for the surface activation. After plasma activation and the subsequent supplying of water as a self-assembly mediate, the chips with the PE-CVD oxide layer were driven by the liquid surface tension and precisely aligned on the host wafers, and subsequently, they were tightly bonded to the wafers through the MCtW oxide-oxide direct bonding technology. Finally, a mechanism of oxide-oxide direct bonding to support the previous models was discussed using an atmospheric pressure ionization mass spectrometer (APIMS).

## 1. Introduction

Although interconnect pitches in microelectronic packaging have scaled by a factor of three to four since the 1970s in industry, Si has approximately scaled by 1000-fold during the same time frame [1]. Therefore, the demand on interconnect scaling will be further enhanced in flip-chip assembly. 3D integration with metal microbumps can address the issue of packaging density, and in addition, parallel signal processing with through-Si vias (TSVs) can realize high-speed and low-power heterogeneous system integration with microelectromechanical systems (MEMS) photonics, passives, and complementary metal-oxide semiconductor (CMOS) toward upcoming internet of things (IoT) societies [2,3,4,5,6]. In recent years, the pitches of solder microbumps with Cu pillars have been significantly decreased down to 10 µm or less [7,8,9]. Good cross-sectional scanning electron microscope (SEM) images showing bonded microbumps have been seen from the previous papers, however, perfect interconnection with millions of microbumps or beyond is still challenging due to poor height uniformity of electroplated solder/Cu microbumps and contamination issues for wafer-level integration. More recently, we have reported a high-yield and high-density Cu-Cu interconnect technology with a sheet of Cu nanopillar based on a MCtW 3D stacking we call reconfigured wafer-to-wafer 3D integration [10].

As is also mentioned in the paper, a known good dies (KGDs) array can be simultaneously and precisely self-assembled on a host wafer by liquid surface tension as a driving force [7,11,12,13]. This methodology can solve a potential tradeoff issue between assembly throughput and assembly accuracies in one-by-one pick-and-place assembly used in conventional chip-to-chip, chip-to-wafer, and chip-to-substrate approaches. Thus, the MCtW integration with the use of KGDs would be expected for advanced packaging including multi-layer chip stacking [14].

Another serious issue is underfilling into small gaps between three-dimensionally stacked chips with fine-pitch microbumps. Pre-underfill materials such as non-conductive paste (NCP) and non-conductive film (NCF) have been developed to solve the issue. However, the NCP technology involves sequential low-throughput dispensing processes, whereas the NCF technology called wafer-level underfilling still requires pick-and-place assembly after dicing of NCF-laminated wafers. We have presented a fully wafer-level underfill technology using a new NCF technology combined with MCtW capillary self-assembly [15,16]. The NCF technology can reduce the pitches of solder/Cu microbump down to 30 µm or less.

Hybrid bonding has also been actively studied to further reduce interconnect pitches. Simultaneous bonding of metals/metals and dielectrics/dielectrics is essential for the technologies. Fine-pitch interconnections using the hybrid bonding technologies have been introduced by several researchers [17,18,19,20,21]. These hybrid bonding methods using plasma surface treatments are processed at temperatures in the range of 200–350 °C in wafer-level processing. Since hybrid bonding technologies are challenged by the momentous tasks of Cu-CMP processes to obtain extremely small surface roughness and total thickness variation (TTV) with low cost and high process controllability, new metal/metal bonding technologies insensitive to the surface topography and uniformity are recommended as described above [10].

On the other hand, oxide-oxide direct bonding is a promising candidate for ultra fine-pitch interconnect formation without metal/metal bonding including microbumps [22,23]. Using the direct bonding technologies, wafers or KGDs can be tightly bonded to the corresponding target wafers without adhesives or underfill resins. The technologies have been traditionally used to fabricate silicon on insulator (SOI) wafers [24]. In addition, no sophisticated CMP processes with heterogeneous materials of metals and dielectrics are required. Ultra fine-pitch TSVs with the pitches of 3 μm [25] can be formed through directly bonded wafers/KGDs with traditional high-resolution photolithography processes, and thereby, upper and lower wafers/KGDs can be electrically connected after direct bonding. In this paper, we employ oxide-oxide direct bonding for self-assembly based MCtW 3D integration, especially for via-last/backside-via 3D stacking. The direct bonding technologies need to be processed at relatively low temperatures with plasma surface activation so as not to apply thermal stress to the devices and wirings of wafers or KGDs [26]. We have previously studied a unique room-temperature pressure-free oxide-oxide direct bonding process combined with surface tension-driven self-assembly using water droplets including a small quantity of hydrogen fluoride [27].

Multichip-to-wafer 3D integration approaches using self-assembly and oxide-oxide direct bonding are classified into two categories: “non-transfer stacking” without support wafers and “transfer stacking” with support wafers which we call Self-Assembly and Electrostatic (SAE) carrier wafers [28]. Since no support wafers are required for the non-transfer stacking based 3D integration, this approach seems to be cost effective and high throughput, but this is not necessarily advisable. Target bottom wafers and lower KGDs receive repeated thermomechanical stresses and damages through the 3D integration processes with thinning and TSV formation, as the number of stacked layers increases. This means that transfer-stacking based 3D integration would be useful for multi-layer 3D chip stacking to increase total production yields. In this paper, however, we focus on 3D stacking of the first layer KGDs on target wafers using oxide-oxide thermocompression direct bonding [29]. Therefore, non-transfer stacking based 3D integration using MCtW self-assembly is adopted.

**Figure 1:**
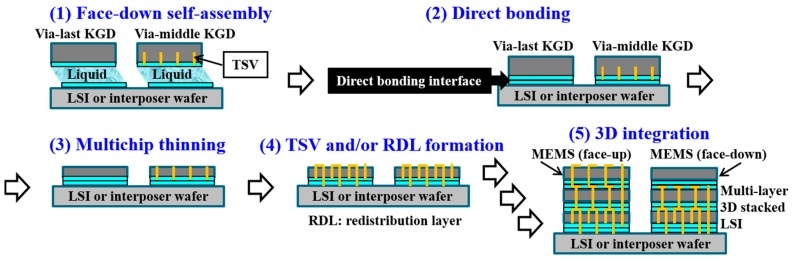
Typical non-transfer stacking based heterogeneous 3D system integration using MCtW self-assembly and thermocompression direct bonding.

Figure 1 schematically shows the total process flow of the non-transfer based heterogeneous 3D integration approach using MCtW oxide-oxide direct bonding with self-assembly. The sequence is also applicable to 3D stacking of KGDs with TSVs fabricated in via-middle processes. First, KGDs with a CMP-treated plasma-CVD SiO_2_ layer on their top are self-assembled in a face-down configuration to a host target wafer on which hydrophilic assembly areas and the hydrophobic background region are formed. Prior to the self-assembly process, plasma activation is performed on the surface of the wafers and/or KGDs. After liquid evaporation, the KGDs are thermally compressed on the wafer, followed by multichip thinning. Then, ultra fine-pitch TSVs are formed on the KGDs. When multi-layer chip stacking is required, the repetition of these process can produce 3D chips with high-density TSVs. When MEMS chips with brittle movement elements and thermally unstable devices are integrated in heterogeneous microelectronic 3D system, it would be suitable for stacking on the top or directly mounted on the Si interposers due to the process compatibility.

## 2. Results and Discussion

### 2.1. Evaluation of Interfacial Energies of Directly Bonded Wafers

Prior to MCtW direct bonding, wafer-to-wafer direct bonding processes are employed to measure oxide-oxide bonding energies of CMP-treated and plasma-activated SiO_2_ used in our experiments. Although a cleavage method with blade insertion is applicable to quantitatively evaluate bonding interface between wafers with beveled edges, typical chips have no bevel parts around their edges, and thus, their bonding interface energies cannot be determined by the method. First of all, the dependences of the bonding energies of directly bonded wafers on oxide types (thermal oxide or PE-CVD oxide) and gaseous activated species (Ar or N_2_) are investigated for comparison to previous works [30,31,32,33,34].

In the experiment, 0.28-mm thick Si wafers with 0.3-µm-thick thermally oxidized (high-purity steam is generated by burning H_2_ and O_2_ gases at 1000 °C) or 1.5-µm-thick PE-CVD deposited SiO_2_ layers were used for direct bonding. The PE-CVD SiO_2_ layers were planarized by SiO_2_-CMP with a ceria-based slurry, resulting in the *R_a_* roughness of 0.2 nm. The as-deposited thermally oxidized wafers and the CMP-treated wafers with PE-CVD SiO_2_ were cleaned with deionized water in a megasonic bath. In the subsequent plasma irradiation process, the surfaces of the wafers were activated with N_2_ or Ar plasma with a widely used sputtering system (CFS-4ES, Shibaura Mechatronics Corporation), followed by dipping in deionized water for 10 min and spin-drying.

In the next bonding process, these wafers were thermally compressed at 300 °C in a vacuum atmosphere of 5 Pa, using a wafer bonder (EV520, EVG). Here, direct bonding experiments were performed under seven different conditions. The top wafers were directly bonded to the bottom wafers with the same oxide types (thermal or PE-CVD oxide). The SiO_2_ surfaces of the top and/or bottom wafers were activated by plasma irradiation using Ar or N_2_ gas.

**Figure 2:**
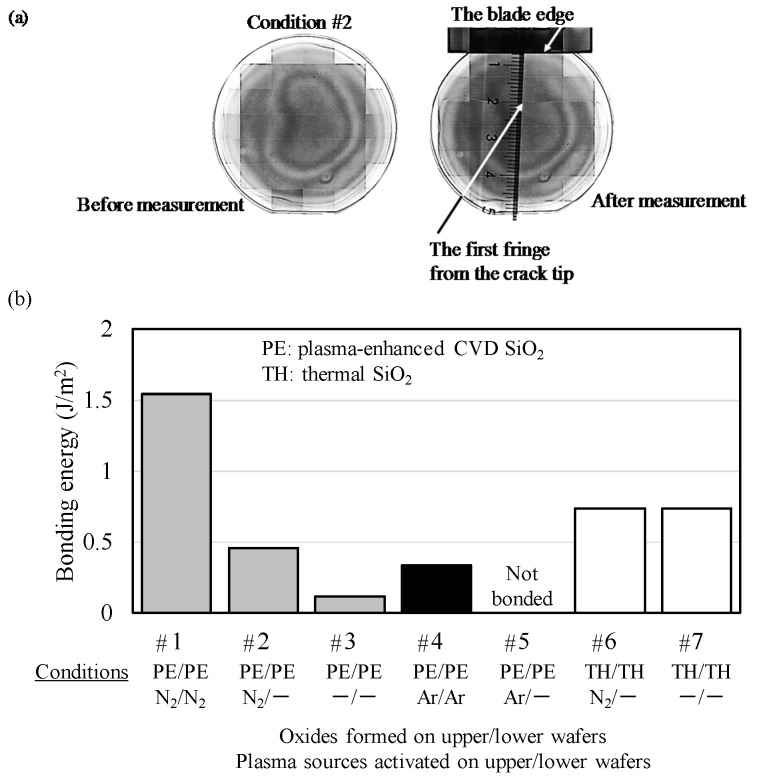
An example of IR images of PE-CVD oxide-oxide bonding (#2) before and after blade insertion test (**a**) and bonding interfacial energies of directly bonded wafers (#1–#7) (**b**).

The bonding strengths of wafer-to-wafer direct SiO_2_ bonding were determined by the fracture toughness (bonding energy) *G*. These bonding energies using the double cantilever beam (DCB) technique were calculated by a crack length that appeared when a blade was inserted into a bonded wafer on the basis of a crack propagation theory as follows [35,36]:

*G* = 3*Et*^3^*y*^2^/4*L*^4^,

where 2*y*, *L*, and *t* are the blade thickness, the crack length from the wafer edge to a bonding/debonding border, and the wafer thickness, respectively. *E* is the Young’s modulus. In this study, the wafer thickness *t* is 0.28 mm, the blade thickness 2*y* is 0.15 mm, and the Young’s modulus of Si<110> is 166 GPa [35]. The crack length *L* was measured by the distance between the first fringe from the crack tip and the actual blade edge using a ruler and IR camera (Infrared Transmission Analysis Inspection System IRise, Moritex Corporation) as shown in Figure 2a. Indeed, when the fringes are observed with the IR light passing through Si, the wafers are separated by a quarter of the observation wavelength (λ/4): the value is typically 250 nm or above (λ is 1 μm at least). We do not take into account the small distance between the first fringe position and the real crack tip position. The measurements were performed within 5 min at 60% relative humidity or more immediately after the blade insertion with a blade speed of more than 600 mm/min. The blade velocity was not precisely controlled. The surface energy γ meaning intrinsic surface energy of one crack surface is generally expressed by dividing the fracture toughness (bonding energy) *G* of bonded wafers by two [37]. Regarding the above-mentioned equation, the errors about 20% or more have to be taken into account, considering a wafer thickness variation around ±20 μm, a crack length accuracy around ±5%, and a blade thickness precision.

**Figure 3:**
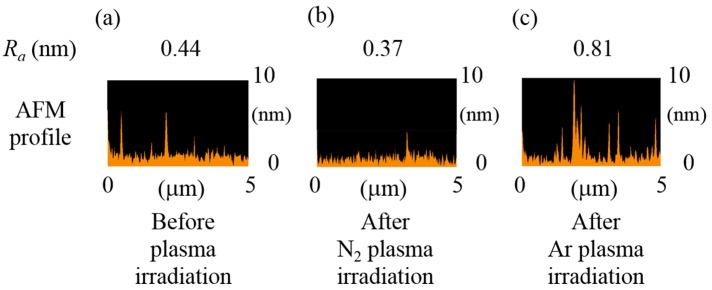
AFM profiles and surface roughness before (**a**) and after plasma irradiation (**b**,**c**) on the surface of CMP-treated PE-CVD oxide.

Figure 2b shows the interfacial bonding energies of bonded wafers prepared by wafer-to-wafer oxide-oxide direct bonding under the various seven conditions. As seen from this figure, the bonding energies of the bonded wafers activated with N_2_ plasma under the conditions #1 and #2 were significantly higher than those of the ones activated with Ar plasma under the conditions #4 and #5. These results indicate that N_2_ plasma is a more effective surface activation species than Ar plasma to obtain high bonding quality. Figure 3 shows atomic force microscopy (AFM) profiles before and after plasma irradiation on the surface of CMP-treated PE-CVD oxide. As seen from this figure, Ar plasma induces large surface roughness of 0.81 nm on the CMP-treated oxide due to the ion bombardment effect of Ar atoms that are heavier than N_2_. The N_2_ plasma keeps the extremely smooth SiO_2_ surface with a roughness *R_a_* of around 0.44 nm after plasma irradiation. The bonding energy obtained from the condition #1 (CMP-treated PE-CVD oxide activated with N_2_ plasma both on upper and lower wafers) is good consistent with that reported in the previous work [33]. In contrast, CMP-treated PE-CVD oxide without plasma activation (the condition #3) shows much lower bonding energy of the previous one [33]. There are no detailed CMP/cleaning conditions and activation power/time in the previous work, but the low bonding energy is due to the total thickness variation of the CMP-treated wafers and the locally high points shown in Figure 3a coming from residual particles such as slurries used in our SiO_2_-CMP process although plasma activation with N_2_ tends to reduce the asperity summits shown in Figure 3b. In other word, the PE-CVD-treated oxide-oxide bonding without plasma activation in this work has high potential to give further high bonding energies by optimizing the CMP/cleaning conditions. In this work, wafer-to-wafer bonding was performed by thermal compression because of the large wafer bow caused by the mechanical stress of the PE-CVD oxide. The measured wafer warpage profile was not shown here, but the value was 25 μm over the 2-inch Si wafer with an as-deposited 1-μm-thick PE-CVD oxide deposited at 350 °C, which would be attributed to the low bonding energies.

On the other hand, the wafers bonded through the thermal oxide layers under the conditions #6 and #7 show quite larger bonding energies than those through the PE-CVD oxide layers under the conditions #2 and #3. The bonding energy of #7 (non-activated thermal oxide bonding) is slightly higher than that in previous direct bonding works [31,33]. However, the bonding energy of #6, thermal oxide bonding activated on one wafer, seems to be approximately half of that reported in the previous work [31]. There is the possibility that the low bonding energies obtained from plasma-activated thermally oxidized wafers bonding in this work are due to the N_2_ conditions such as plasma power, activation time, and/or organic contamination from the sputtering equipment used in this plasma activation process. Since the quick blade insertion in this work has been carried out at high humidity atmosphere although the bonding energy measurement has been done within 5 min directly after the blade stop, the resulting bonding energies could show lower values due to external water stress corrosion during the DCB measurement [34], especially for low-power deposited PE-CVD oxides sensitive to moisture [38].

### 2.2. Evaluation of Bonding Strengths of Directly Bonded Chips

MCtW direct bonding is demonstrated and the shear bonding strengths of the chips assembled on wafers are measured using thermal SiO_2_ or CMP-treated PE-CVD deposited SiO_2_ with N_2_ plasma activation and the subsequent water dipping processes. In the experiments, 0.28-mm thick wafers and chips with a size of 5 mm × 5 mm were used. The wafers were treated with N_2_ plasma, and subsequently dipped into deionized water and spin-dried. In contrast, the non-activated chips were cleaned with a H_2_SO_4_/H_2_O_2_/H_2_O solution to remove organic contaminants prior to assembly. The wafers were set on the lower stage of a wafer bonder EV 520, and the chips were assembled on the wafers in a face-down configuration. After that, a compliant soft graphite sheet and an additional carbon plate were placed on the top of the chip array. Because the graphite sheet is a deformable material, the sheet acts as a TTV absorber to provide uniform compressions for all the KGD chips on the corresponding wafers. In addition, the graphite sheet has high thermal conductivity, and thereby, the heating temperature can be well controlled during the MCtW bonding process. In the direct bonding, the chips were thermally compressed to the corresponding wafers through the graphite sheet at 300 °C in a vacuum atmosphere of 5 Pa with a bonding pressure of 0.8 N/chip or 10 N/chip. After the direct bonding process, the shear bonding strengths between the chips and the wafers were measured with a bond tester.

Table 1 shows the bonding yields of the chips simultaneously and directly bonded to N_2_ plasma-assisted wafers under the three conditions. The bonding yield is determined by the ratio of the number of tightly bonded chips that withstand 5-kg shear load to total number of measured 12 chips. The chips with share bonding strengths of below approximately 2 MPa are defined as non-bonded chips. As seen from the table, the bonding yield in the low bonding pressure of 0.8 N/chip was much lower than that of the high bonding pressure of 10 N/chip. The MCtW direct bonding between thermal oxide layers shows a good yield, exceeding 90% when the bonding pressure of 10 N/chip was used. The reason why the yield has not reached 100% is probably due to particles resulted from the experimental environment and/or the chipping defects of chip edges. In contrast, the yields are approximately 30% when the pressure of 0.8 N/chip was used for MCtW direct bonding with thermal oxides. Therefore, high bonding pressure above 10 N/chip seems to be appropriate to stably obtain the thermocompression direct bonding yields beyond 90%. The low bonding pressure of 0.8 N/chip would provide the chip array and the wafers with non-uniform thermal compression, because this pressure might not be sufficient to completely deform the graphite sheet.

**Figure 4:**
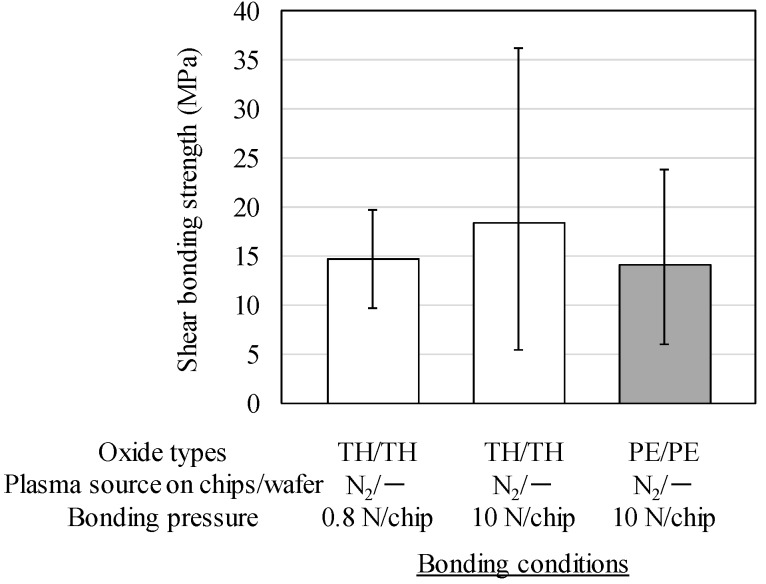
Shear bonding strengths of chips directly bonded to wafers with N_2_ plasma activation.

Figure 4 shows the shear bonding strengths between the chips and the wafers of the three conditions described above. As shown in this figure, the low bonding pressure of 0.8 N/chips resulted in a relatively high bonding strength of 15 MPa although the bonding yield was low. On the other hand, the average shear bonding strengths with the high bonding pressure of 10 N/chip were 18.3 MPa and 14.0 MPa for thermal oxide and CMP-treated PE-CVD oxide, respectively, as shown in this figure. These results show that the shear bonding strengths between the chips/wafers with the PE-CVD oxide layer were comparable to those with the thermal oxide layer. This tendency is similar to that observed in the wafer-to-wafer direct bonding experiment described in the previous section of #2 and #6 in Figure 2b. These resulting bonding strength of PE-CVD oxide-oxide bonding in Figure 4 is high enough to endure the back side grinding and CMP processes during multichip thinning. These results give a positive prospect for the application of the MCtW oxide-oxide direct bonding technologies for 3D integration. In addition, these advanced direct bonding technologies can be expected to further progresses in via-last/backside-via TSV formation, because the thermally stable bonding interface is required to the repeated 3D stacking processes.

The effect of both the N_2_ plasma activations and water dipping processes on the shear bonding strengths is evaluated. Three types of surface treatments were employed: the first condition was plasma activation without water dipping water. The second condition was water dipping without plasma activation and the third one was plasma activation and the subsequent water dipping. The plasma activation with N_2_ gas was performed with a widely-used sputtering system (SH-550, ULVAC). After the MCtW direct bonding processes, the shear bonding strengths of the directly bonded chips were measured with the bond tester.

**Figure 5:**
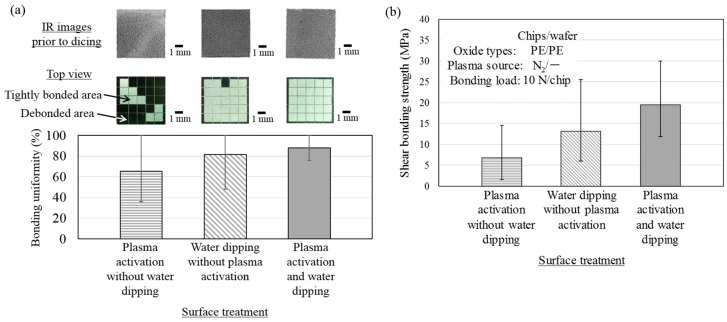
Bonding uniformity of chips directly bonded to wafers conditioned with different surface treatments (**a**). The IR images of chips are taken after MCtW direct bonding prior to dicing and the top views show a whole chip after dicing. The error bars indicate the max. and min. ratio of “the number of non-debonded 1-mm-square small dielets per a 5-mm-square chip” to “25 (the total number of the small dielets after dicing per the chip)”. Shear bonding strengths of chips directly bonded to wafers conditioned with different surface treatments (**b**).

Next, in order to evaluate the in-plane uniformity of the bonding strengths of a chip directly bonded to the corresponding wafers under these conditions, dicing tests were performed. In the dicing tests, the 5-mm-square chips directly bonded on the wafers were further singulated into 1-mm-square dies with a saw dicer, and the number of the remaining dies against the dicing stress were counted for evaluating bonding uniformity. Here, we used 3 chips for each direct bonding condition, so totally 75 pieces of 1-mm-square dies are obtained. Figure 5a shows an IR image and a photomicrograph of a directly bonded chip before and after cutting into the 1-mm^2^ dies, respectively. Unbounded areas or entrapped particles can be easily observed by the IR images, but the bonding strengths of the bonded areas are not figured out. In this figure, the percentage of the 1-mm^2^ dies that are remained bonded after the dicing process represents the bonding uniformity of the chips directly bonded through the CMP-treated PE-CVD oxide under the three surface treatment conditions. The bonding uniformity obtained by the dicing method is not necessarily quantitative. However, this method was employed to compare among various conditions of surface treatments for oxide-oxide direct bonding [39]. As shown in the figure, the bonding uniformity decreases with the used surface treatment in the following order: plasma activation with water dipping, water dipping without plasma activation, and plasma activation without water dipping. These results suggest that N_2_ plasma irradiation not only helps to strengthen the MCtW direct bonding with PE-CVD oxide, but also increases the bonding yields evaluated by dicing.

Figure 5b shows the shear bonding strengths of the directly bonded chips using the three types of surface treatments. The strongest average shear bonding strength was 19.5 MPa, and was obtained with both the surface treatment of plasma activation and water dipping. The surface treatments with plasma activation exhibited average direct bonding strengths of 6.8 MPa, whereas the water dipping treatment exhibited 13.1 MPa. These results indicate that the wafer surface pre-treatment by water dipping makes a strong contribution to the achievement of the high bonding strengths for the MCtW direct bonding processes, compared to N_2_ plasma irradiation. As seen from Figure 5a,b, water absorption on the surface and/or subsurface [31] of the host wafers would act as a dominant factor to obtain both high bonding strengths and high bonding uniformity on the entire surface of the chips.

### 2.3. Self-Assembly of Chips on Wafer

**Figure 6:**
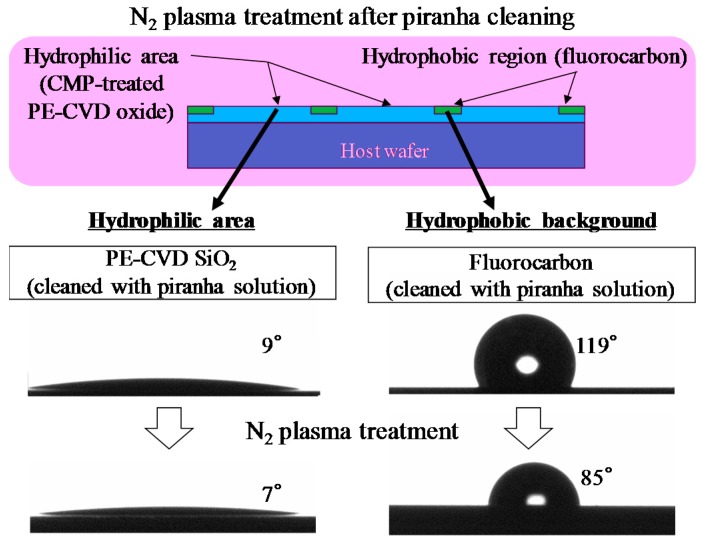
Water contact angles before and after N_2_ plasma activation on a hydrophilic assembly area, and hydrophobic background region.

**Figure 7:**
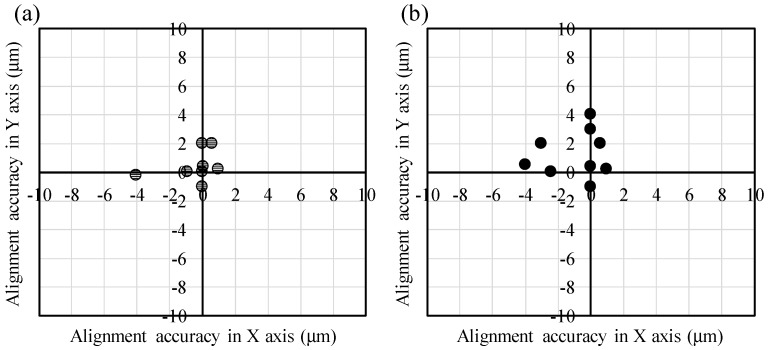
Alignment accuracies of self-assembled chips before and after thermal compression of MCtW direct bonding.

In the “non-transfer stacking” scheme, plasma activation is applied only on chips to be self-assembled because the N_2_ plasma treatment decreases the hydrophobicity of the regions surrounding the assembly areas. The water contact angle is reduced from 119° to 85° after N_2_ plasma activation, as shown in Figure 6. When the low hydrophobic surrounding region is employed for self-assembly, the alignment accuracies are significantly decreased. Therefore, plasma activation is applied only on the surface of chips prior to self-assembly. The chips with CMP-treated PE-CVD oxide are self-assembled upside down to the host wafer. As shown in Figure 7a, almost all the chips exhibit high alignment accuracies within approximately 2 µm even when these chip are manually positioned at the release point higher than the surface of the droplets by 1 mm or more with coarse *X*/*Y*/θ pre-alignment. The average alignment accuracies of all self-assembled chips before direct bonding are −0.36 µm and 0.27 µm with a standard deviation of 1.46 µm and 1.10 µm in *x* and *y* directions, respectively. These accuracies include systematic errors caused by wafer tilt and *xy* sift in photolithography processes to define the size of chips and assembly areas. The size shrinkage of chips used in this study were within 2 µm that was resulted from the excess side etching under the plasma dicing conditions for fabricating thick chips with a thickness of 0.28 mm. In addition, the outer size patterns of the 5-mm-square chips and micro-vernier patterns formed on the chips to evaluate self-alignment accuracies were determined by a mask aligner that empirically gives alignment error of ±2 µm at most. However, misalignment of photomasks generally includes not only *xy* errors but also theta error, and thus, the potential maximum self-assembly error cannot be precisely defined. On the other hand, the average alignment accuracies and standard deviations with absolute numbers in *x*/*y* directions are 0.73 µm /0.76 µm and 1.29 µm /0.80 µm for all self-assembled chips before direct bonding, respectively. The relatively large statistics are explained by the local minimum energies that are present in this capillary chip self-assembly system. Ideally, a chip would be positioned to the center of the host hydrophilic assembly area by self-assembly, otherwise the chip would be positioned to the edges of the hydrophilic area. On the way to final positioning in self-assembly processes, the chips are often fixed due to the chip own weight against restoring forces driven by water surface tension and/or friction force from host wafers (hydrophobic surfaces) due to the chip tilt. The big statistics is also mainly attributed to the manual chip handling (not well controlled chip pre-positioning), giving large initial offsets in *x*/*y*/*z*/θ directions in addition to chip tilt prior to chip landing onto the top of the water droplets immediately after chip release [40,41,42,43]. We can further increase the alignment accuracies and reduce the variation by chip thickness reduction down to 100 μm or below, by the use of automatic/robotic chip handling systems, and by optimization of lithographic conditions using steppers to define the vernier positions toward outer sizes of the corresponding hydrophilic assembly areas.

**Figure 8:**
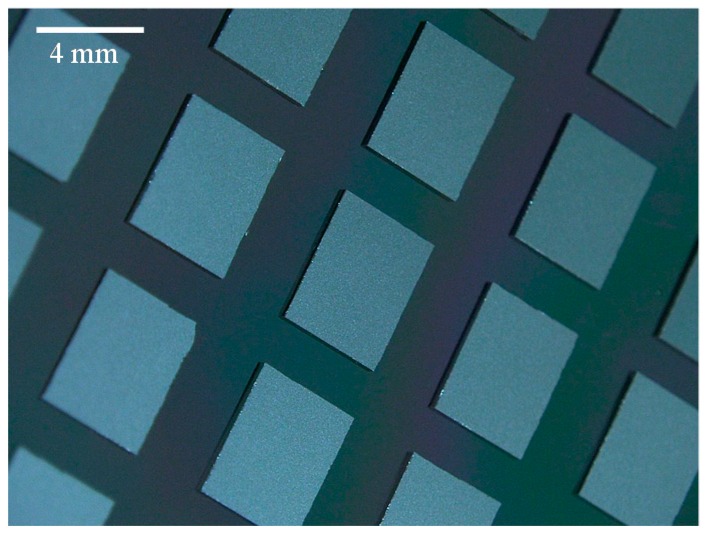
A photomicrograph of self-assembled chips directly bonded to a wafer through CMP-treated PE-CVD oxide.

The following oxide-oxide direct bonding is curried out with the EV520 bonder to provide thermal compression through the flexible graphite sheet used as a TTV absorber for uniform compression of the self-assembled chips on the host wafers. As shown in Figure 8, these chips are precisely aligned and bonded on wafer when the high hydrophobic surrounding region are employed. Finally, these chips are annealed at 300 °C in vacuum under 5 Pa with a bonding load of 10 N/chip. Prior to the thermal compression process, the self-assembled chips are not tightly bonded on the wafer through Van der Waals interaction between the oxide surface and water used as a self-assembly mediate. As shown in Figure 7b, the alignment accuracies are slightly degraded due to the chip shift by the compressive force. The average alignment accuracies of all self-assembled chips after direct bonding in *x* and *y* directions are 1.22 and 1.46 µm with a standard deviation of 1.78 and 1.61 µm, respectively. The slight chip shift by thermal compression would be restricted by transfer-stacking based MCtW 3D integration with SAE carrier technologies in which self-assembled KGDs are temporarily fixed by electrostatic adhesion [28]. The yields of successful chip bonding are nearly 100% when we use 10 N/chip in bonding load. The resulting shear bonding strengths are 16.0 MPa on average for the CMP-treated chips conditioned with plasma activation and water dipping treatments. It is said that the effect of water dipping is much higher than that of plasma activation. These bonding strengths are sufficiently high enough to allow backside grinding and Si-CMP during multichip thinning.

### 2.4. Impact of Plasma Activation on Oxide-Oxide Direct Bonding

The effects of N_2_ plasma irradiation and water dipping on MCtW direct bonding are characterized in this session. Therefore, most papers have discussed oxide-oxide bonding with thermally oxidized Si wafers and/or bare Si wafers in detail [30,31,44,45]. A few papers have described the detailed mechanism of direct bonding through CVD oxide [34,38].

**Figure 9:**
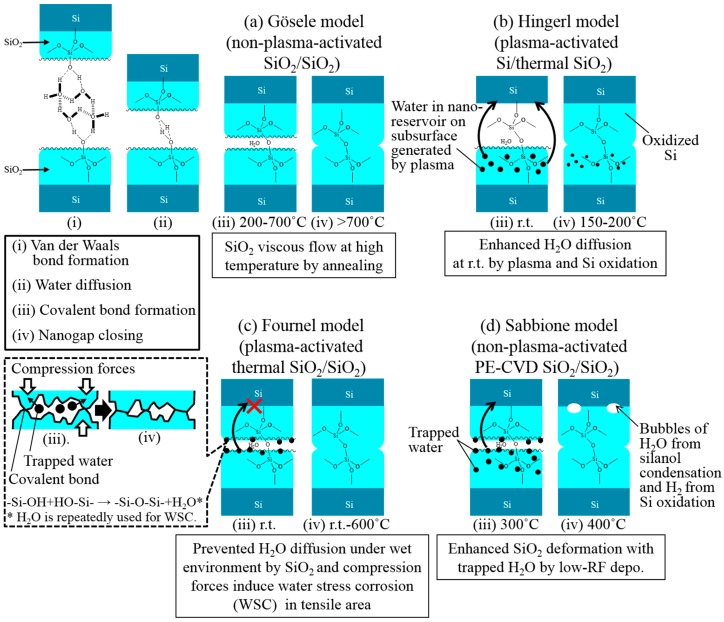
Four models of Si/SiO_2_ and SiO_2_/SiO_2_ direct wafer bonding.

Gösele et al. have reported that the chemistry of the Si–Si bonding process with wafers is basically the same as in the case of fused silica, since bare Si wafers are normally covered with at least a few angstroms of native oxide [30]. In the Gösele model, Van der Waals forces based on hydrogen bond networks between oxygen and hydrogen atoms dominates SiO_2_–SiO_2_ direct bonding through absorbed water layers in which water molecules form a water cluster at the temperature of below 100 °C. As the temperature goes up at above 100 °C, the water can diffuse around the bonding interface, resulting in hydrogen bonds between silanol groups on the surface of upper and lower wafers. Then, covalent bonds of siloxane linkages are formed at the SiO_2_–SiO_2_ bonding interface at temperatures ranging from 200 °C to 700 °C. It is not until the temperature is increased beyond 700 °C that the SiO_2_–SiO_2_ interface permits closure of nano-gaps between the Si wafers. This mechanism of SiO_2_ viscous flow at high temperature by annealing is illustrated in Figure 9a.

On the other hand, a model for plasma-activated oxide-oxide direct bonding of Si wafers has been recently proposed by Hingerl et al. [31]. They have described that the creation of subsurface defects (nano-reservoir) is caused by surface plasma irradiation and that the reservoirs contribute to the high surface energies of the directly bonded wafers. Figure 9b shows a schematic drawing explaining the nanogap closing mechanism on plasma-assisted direct bonding of Si wafers. Direct bonding with a thermally oxidized wafer and a bare Si wafer with a very thin native oxide layer is assumed in this model. As shown in the figure, the water trapped in the nano-reservoirs diffuses to Si bulk through a thin native oxide layer and oxidizes the Si to close the nanogap between the wafers by minimizing the sum of the free surface energy and strain energy in the oxide. By using the plasma surface activation, ≡Si–O–Si≡ covalent bonds would be not only formed at room temperature but also the closure of nanogaps could take place at around 200 °C. Although Hingerl et al. have not referred to their detailed SiO_2_/SiO_2_ bonding mechanism [31], the bonding energy seems to be lower than that of Si/SiO_2_. Perhaps, this is because thick oxide layer prevents the diffusion of the water molecules into the Si bulk.

Fournel et al. have reported SiO_2_/SiO_2_ bonding mechanism where the difference between Si/SiO_2_ and SiO_2_/SiO_2_ interfaces for plasma-activated direct bonding is described [34]. They have proposed roughness asperity evolution with internal water stress corrosion for plasma-activated Si/SiO_2_ or SiO_2_/SiO_2_ direct bonding. In the Fournel model, as shown in Figure 9c, the interfacial water is consumed during low-temperature Si oxidation that starts around 150 °C, which means no remaining water at the Si/SiO_2_ interface. In contrast, SiO_2_/SiO_2_ interfaces prevent the interfacial water from diffusing through the thick SiO_2_ layers of a 145-nm-thick thermal oxide. Therefore, the remaining water between contacted asperities penetrates the tensile SiO_2_ regions induced by compression forces, and then, the small gaps are closed by the internal water stress corrosion at the SiO_2_/SiO_2_, resulting in high bonding energy.

**Figure 10:**
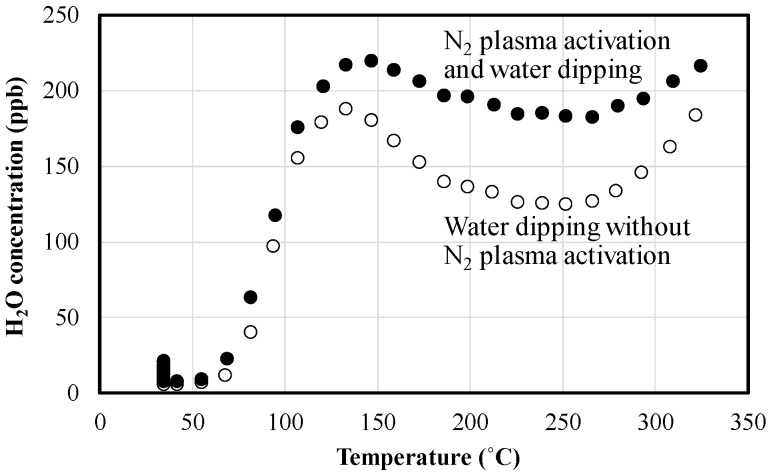
Water storage properties of PE-CVD SiO_2_ layers with and without N_2_ plasma irradiation.

In this work, to analyze the mechanism of plasma-assisted oxide-oxide direct bonding with a water dipping process, the water storage properties of CMP-treated PE-CVD SiO_2_ surfaces with and without N_2_ plasma irradiation were quantitatively measured with an APIMS. APIMS enables us to know the quite small amount of vaporized H_2_O molecules desorbed from wafer surfaces at atmospheric pressure [46]. Compared with thermal desorption spectroscopy (TDS), APIMS can precisely detect H_2_O concentrations because almost all H_2_O molecules are desorbed from the surface of the wafers prior to measurement at a desired temperature region under the high-vacuum conditions of TDS. For the APIMS measurement, 0.28-mm thick Si wafers with a PE-CVD SiO_2_ layer were prepared. The wafers were treated with/without N_2_ plasma, and then, dipped into deionized water and subsequently spin-dried. After that, the wafers were set into an APIMS system and H_2_O concentrations were detected. Figure 10 shows the water storage properties on the PE-CVD SiO_2_ treated with/without N_2_ plasma. In the figure, the *x*- and *y*-axis indicate measurement time and H_2_O concentrations. 2498 ng of H_2_O molecules were detected with a non-activated wafer with PE-CVD oxide, whereas 3304 ng of H_2_O molecules were detected with the plasma-activated wafer with the oxide layer. As seen from these results, approximately 1.3-fold H_2_O molecules are stored on/in the PE-CVD SiO_2_ treated with N_2_ plasma, compared with that without the plasma treatment. The water molecules stored on the surface of the PE-CVD SiO_2_ layer are dramatically increased at above 70 °C. These results show that plasma activation is effective to obtain high-water-content surfaces. It can be said that the amount of absorbed water molecules on the surface of the oxide is increased by the plasma activation. There is also the possibility that the nano-reservoirs generated by the plasma irradiation process could contribute to the water storage property of SiO_2_ surfaces as described by Hingerl et al. [31]. As reported by Fournel et al. [34], however, if water stress corrosion is happened at the plasma-activated PE-CVD SiO_2_/SiO_2_ interface, the high bonding energy under the condition #1 against #3 listed in Figure 2 is good agreement with their model dealing with internal water stress corrosion that induces high bonding energies. On the other hand, Sabbione et al. [38] have expatiated on the PECVD oxide-oxide direct bonding in which absorbed water in the oxide reduces the hardness as a mechanical property, leading to an increase in local bonding area due to the enhanced elastic deformation. In addition, low RF power of the PE-CVD oxide deposition gives a decrease in the modulus and hardness as well. They also have demonstrated that the absorbed water diffuses through a 400-nm-thick PE-CVD oxide and reaches Si bulk, followed by oxidation at 400 °C although plasma activation is not employed. Considering these previous works, it will be noted that water molecules play a crucial role for oxide-oxide direct bonding. As seen from the results obtained under the conditions #1 and #3 in Figure 2, our thermocompression oxide-oxide bonding would be affected by the internal water stress corrosion. In this study, it is not clear whether the silanol condensation and oxidation reactions are occurred at the Si/SiO_2_ interface as shown in Figure 9d. However, our oxide-oxide thermocompression direct bonding is actually enhanced by “water” trapped on the surface (and/or subsurface) of and the inside of the PE-CVD oxide deposited at relatively lower RF. As seen from the APIMS analysis of the plasma-activated PE-CVD oxide, the water uptake quantity is undoubtedly high. As is also seen from the AMF profiles in Figure 3a,b, the asperity summits must be reduced by the plasma treatment. The elastic deformation of our PE-CVD oxides would be supported by the thermocompression bonding although Sabbione et al. have described small difference in bonding energy between thermocompression and load-free simple annealing [38]. The other parameters such as CMP/cleaning conditions, internal stress, the amount of Si–OH content on the surface of CMP-treated PE-CVD oxides, and the deposition temperature would impact on the thermocompression direct bonding energies. For these reasons, a comprehensive understanding of oxide-oxide bonding mechanisms warrants separate studies on PE-CVD chemistries.

## 3. Experimental Section

### 3.1. Chip/Wafer Fabrication

Chips and wafers used for self-assembly in this study were fabricated with 100-nm-thick thermally oxidized p-type Si(100) wafers with a thickness of 0.28 mm. In the chip fabrication process, micro-vernier patterns were formed on 1-µm-thick PE-CVD oxide with 100-nm-thick sputtered Al by standard *i*-line photolithography with a mask aligner (MA8, SUSS MicroTec.) and the subsequent wet etching. The PE-CVD oxide was formed with tetraethyl orthosilicate (TEOS) at 350 °C in this study. The RF power of the PE-CVD system was 300 W. Then, an additional 2-µm-thick PE-CVD oxide layer was planarized by SiO_2_-CMP with a table-top high precision lapping machine (NF-300, Nanofactor) using a CMP pad of IC1000 (Nitta Haas Inc.) and a ceria-based slurry system consisting of CeO_2_ (HS-8005) and additives (HS-8102GP), kindly supplied from Hitachi Chemical Co., Ltd., followed by cleaning with PC07B (Stellla-Chemifa Co.) and water without ultrasonication. After that, plasma dicing was implemented with Bosch etching to obtain the 0.28-mm-thick chips to be self-assembled: the outer sizes of chips were also determined by the *i*-line photolithography technique. The resulting chip size was 4 mm × 5 mm. On the other hand, the procedures of micro-vernier pattern formation and SiO_2_-CMP on host wafers were the same to the chip fabrication. After the SiO_2_-CMP process, recess etching with a depth of 10 μm was performed on the planarized wafers. Finally, a 10-nm-thick fluorocarbon material was deposited on the wafers by a lift-off technique with acetone, resulting in an array of hydrophilic SiO_2_ assembly areas and a hydrophobic fluorocarbon background region.

### 3.2. Self-Assembly

**Figure 11:**
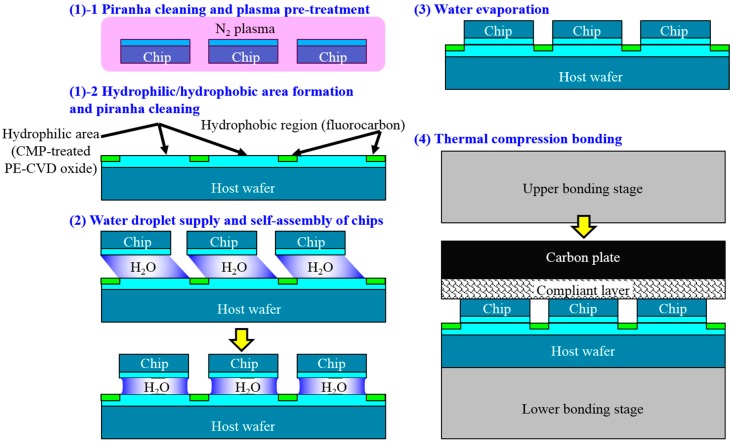
A process flow of MCtW self-assembly and direct bonding.

Self-assembly with the chips and the host wafers are schematically shown in Figure 11. The chips fabricated by plasma dicing is cleaned with a solution of H_2_SO_4_/H_2_O_2_/H_2_O and then pre-treated with N_2_ plasma using a widely-used sputtering system (SH-550, ULVAC) prior to self-assembly. The plasma activation power is 180 W and activation time is 1 min. On the other hand, the wafers were pre-treated without plasma irradiation: the wafers were only cleaned with the piranha solution (H_2_SO_4_/H_2_O_2_/H_2_O) prior to self-assembly. Then, a microliter of pure water was supplied to each hydrophilic assembly area. After that, the chips with the hydrophilic oxide layer on their front-sides were coarsely pre-aligned with tweezers and placed upside down to the surfaces of the water droplet by hand. Immediately after chip release, the chips were precisely aligned to the pre-determined assembly areas formed on the host wafers. Finally, the water droplets were evaporated at room temperature to give MCtW structures in which the chips and wafers were bonded through hydrogen bond networks with the absorbed water molecules.

### 3.3. Contact Angle

The contact angle was measured on an optical contact angle meter (CAM101, KSV Instruments Ltd.) using a droplet of ultrapure water with a volume of approximately 1 μL by means of the sessile drop method. The droplet was monitored by a high resolution charge-coupled device (CCD) camera and analyzed by Drop Shape Analysis software (CAM 200, KSV Instruments Ltd.) utilizing a monochromatic light source which generates the highest quality images with minimal sample heating. The complete profile of the droplet was fitted by the Young-Laplace equation to give the slope at the three-phase boundary where a liquid, gas, and solid intersect. The resulting static contact angles were determined both at the right and left sides.

### 3.4. Bonding Strength

The shear bonding strength test was carried out with a multi-purpose bond tester (4000Plus, Dage Co.). DS100KG and SHR-250-3000 were used as a load cell and a shear tool: the length of the tip of the tool was 3 mm. Measurement conditions were 100 μm/s in shear speed and 20 μm in shear height.

### 3.5. Alignment Accuracy

Alignment accuracies were evaluated by the observation of micro-vernier patterns formed on the surfaces of chips/wafers on an IR microscope (MX61L, Olympus) with an IR camera (ORCA-Flash4.0 V2 C11440-22C, Hamamatsu Photonics). The resolution is 2048 × 2048 pixels and the pixel size is 6.5 μm × 6.5 μm. The IR optical system gave space resolutions of 0.13 μm × 0.13 μm for 50-power objective lens. The measurement resolution of the micro-vernier patterns is 0.2 μm.

### 3.6. Atomic Force Microscopy (AFM)

Surface roughness of Si substrates was characterized by AFM (Nano Cute, Seiko Instruments Inc.). The AFM topography images were operated in tapping mode by the use of phosphorous doped Si (1–10 Ω·cm) cantilevers. AFM images were recorded on a maximum scan region of 5 μm × 5 μm and with 512 lines.

### 3.7. Atmospheric Pressure Ionization Mass Spectrometer (APIMS)

Plasma-activated and non-activated specimens were prepared on 2-inch wafers with a CMP-treated 2-μm-thick PE-CVD oxide layer. The desorption of H_2_O molecules from the wafers were detected with an atmospheric pressure ionization mass spectrometer (APIMS) (UG-410P, Nippon API). The specimens were thermally annealed with a quartz glass furnace and an IR ray lamp. The measurement conditions were as follows: carrier gas was Ar, flow rate of Ar was 1 L/min, temperature range was 23 °C r.t. to 320 °C, and heating rate was 10 °C/min.

## 4. Conclusions

In this study, we demonstrated plasma- and water-assisted MCtW oxide-oxide thermocompression direct bonding at 300 °C using surface tension-driven self-assembled chips, and evaluated the effect of plasma activation and the subsequent water dipping processes on bonding properties (strength, yield, and uniformity). N_2_ plasma was found to be more effective activation species for this direct bonding technology with PE-CVD oxide than Ar plasma. In addition, the bonding strengths obtained with PE-CVD oxide assisted by N_2_ plasma and absorbed water were comparable to that obtained with thermal oxide in MCtW direct bonding process. We have not yet determined the presence of water reservoirs like nano- or micro-pores. However, it was noteworthy that water supply onto the surface and/or subsurface of the wafers was essential for high-strength and high-uniformity MCtW oxide-oxide direct bonding in addition to N_2_ plasma assistance. The plasma activation is effective for obtaining the high-water-content surfaces on PE-CVD oxide, which is considered as an important parameter to obtain high bond strengths between the chips and the host wafers. This approach consisting of direct bonding with self-assembly can be expected for multichip grinding and TSV liner dielectric deposition in high-throughput and high-yield MCtW 3D integration with high-density via-last/back-via TSV formation due to the mechanically and thermally stable oxide-oxide bonding interface.

## Figures and Tables

**Table 1:** Effect of oxide types and bonding load on bonding yields of chips directly bonded to wafers with N_2_ plasma activation on chips.

**Bonding Conditions**	TH/TH	TH/TH	PE/PE
	N_2_/—	N_2_/—	N_2_/—
	0.8 N/chip	10 N/chip	10 N/chip
**Bonding Yield (%)**	33	92	92

## References

[1] Iyer S.S. (2015). Three-dimensional integration: An industry perspective. MRS Bull..

[2] Matsumoto T., Kudoh Y., Tahara M., Yu K.-H., Miyakawa N., Itani H., Ichikizaki T., Fujiwara A., Tsukamoto H., Koyanagi M. Three-Dimensional Integration Technology Based on Wafer Bonding Technique Using Micro-Bumps. Proceedings of the 1995 International Conference on Solid State Devices and Materials (SSDM).

[3] Ramm P., Bollmann D., Braun R., Buchner R., Cao-Minh U., Engelhardt M., Errmann G., Grassl T., Hieber T.K., Hubner H. (1997). Three dimensional metallization for vertically integrated circuits. Microelectron. Eng..

[4] Koyanagi M., Kurino H., Lee K.W., Sakuma K., Miyakawa N., Itani H. (1998). Future system-on-silicon LSI chips. IEEE Micro.

[5] Koyanagi M., Nakamura T., Yamada Y., Kikuchi H., Fukushima T., Tanaka T., Kurino H. (2006). Three-dimensional integration technology based on wafer bonding with vertical buried interconnections. IEEE Trans. Electron Devices.

[6] Lee K., Noriki A., Kiyoyama K., Fukushima T., Tanaka T., Koyanagi M. (2011). Three-dimensional hybrid integration technology of CMOS, MEMS, and photonics circuits for optoelectronic heterogeneous integrated systems. IEEE Trans. Electron Devices.

[7] Fukushima T., Iwata E., Ohara Y., Murugesan M., Bea J., Lee K., Tanaka T., Koyanagi M. (2012). Multichip-to-wafer three-dimensional integration technology using chip self-assembly with excimer lamp irradiation. IEEE Trans. Electron Devices.

[8] Kondo T., Takazawa N., Takemoto Y., Tsukimura M., Saito H., Kato H., Aoki J., Kobayashi K., Suzuki S., Gomi Y. (2016). 3-D-stacked 16-mpixel global shutter CMOS image sensor using reliable in-pixel four million microbump interconnections with 7.6-μm pitch. IEEE Trans. Electron Devices.

[9] Ohyama M., Nimura M., Mizuno J., Shoji S., Tamura M., Enomoto T., Shigetou A. Hybrid Bonding of Cu/Sn Microbump and Adhesive with Silica Filler for 3D Interconnection of Single Micron Pitch. Proceedings of the Electronic Components and Technology Conference (ECTC).

[10] Lee K.-W., Bea J.-C., Fukushima T., Suresh R., Wu X., Tanaka T., Koyanagi M. (2016). Novel hybrid bonding technology using ultra-high density Cu nano-pillar for exascale 2.5D/3D integration. IEEE Electron Device Lett..

[11] Fukushima T., Yamada Y., Kikuchi H., Koyanagi M. New Three-Dimensional Integration Technology Using Self-Assembly Technique. Proceedings of the IEEE International Electron Devices Meeting.

[12] Fukushima T., Iwata E., Ohara Y., Murugesan M., Bea J.-C., Lee K.-W., Tanaka T., Koyanagi M. (2011). Multichip self-assembly technology for advanced die-to-wafer 3-D integration to precisely align known good dies in batch processing. IEEE Trans. Compon. Packag. Manuf. Technol..

[13] Fukushima T., Bea J.C., Kino H., Nagai C., Murugesan M., Hashiguchi H., Lee K.-W., Tanaka T., Koyanagi M. (2014). Reconfigured-wafer-to-wafer 3D integration using parallel self-assembly of chips with Cu-SnAg microbumps and a NCF. IEEE Trans. Electron Devices.

[14] Sakuma K., Skordas S., Zitz J., Perfecto E., Guthrie W., Guerin L., Langlois R., Liu H., Ramachandran K., Lin W. Bonding Technologies for Chip Level and Wafer Level 3D integration. Proceedings of the 64th Electronic Components and Technology Conference (ECTC).

[15] Ito Y., Fukushima T., Lee K.-W., Choki K., Tanaka T., Koyanagi M. Direct Multichip-to-Wafer 3D Integration Technology Using Flip-Chip Self-Assembly of NCF-Covered Known Good Dies. Proceedings of the 64th Electronic Components and Technology Conference (ECTC).

[16] Ito Y., Murugesan M., Kino H., Fukushima T., Lee K.-W., Choki K., Tanaka T., Koyanagi M. Development of Highly-Reliable Microbump Bonding Technology Using Self-Assembly of NCF-Covered KGDs and Multi-Layer 3D Stacking Challenges. Proceedings of the IEEE 65th Electronic Components and Technology Conference (ECTC).

[17] Goto M., Hagiwara K., Iguchi Y., Ohtake H., Saraya T., Higurashi E., Toshiyoshi H., Hiramoto T. (2014). 3-D silicon-on-insulator integrated circuits with NFET and PFET on separate layers using Au/SiO_2_ hybrid bonding. IEEE Trans. Electron Devices.

[18] Enquist P., Fountain G., Petteway C., Hollingsworth A., Grady H. Low Cost of Ownership Scalable Copper Direct Bond Interconnect 3D IC Technology for Three Dimensional Integrated Circuit Applications. Proceedings of the IEEE International Conference on 3D System Integration (3DIC).

[19] Aoki M., Furuta F., Hozawa K., Hanaoka Y., Kikuchi H., Yanagisawa A., Mitsuhashi T., Takeda K. Fabricating 3D Integrated CMOS Devices by Using Wafer Stacking and Via-Last TSV Technologies. Proceedings of the IEEE International Electron Devices Meeting (IEDM).

[20] Sanchez L., Bally L., Montmayeul B., Fournel F., Dafonseca J., Augendre E., Cioccio L.D., Carron V., Signamarcheix T., Taibi R. Chip to Wafer Direct Bonding Technologies for High Density 3D Integration. Proceedings of the IEEE 62nd Electronic Components and Technology Conference (ECTC).

[21] Mermoz S., Sanchez L., Cioccio L.D., Berthier J., Deloffre E., Coudrain P., Fretigny C. High Density Chip-to-Wafer Integration Using Self-Assembly: On the Performances of Directly Interconnected Structures Made by Direct Copper/Oxyde Bonding. Proceedings of the IEEE 15th Electronics Packaging Technology Conference (EPTC 2013).

[22] Moriceau H., Rieutord F., Fournel F., Imbert B., Cioccio L.D., Baudin F., Rauer C., Morales C. Low Temperature Direct Bonding Assisted by CMP and Plasma Activation. Proceedings of the IEEE 3rd International Workshop on Low Temperature Bonding for 3D Integration (LTB-3D).

[23] Batra P., Skordas S., LaTulipe D., Winste K., Kothandaraman C., Himmel B., Maier G., He B., Gamage D.W., Golz J. (2014). Three-dimensional wafer stacking using Cu TSV integrated with 45 nm high performance SOI-CMOS embedded DRAM technology. J. Low Power Electron. Appl..

[24] Sugimoto F., Arimoto Y. (1992). Bond strength of bonded SOI wafers. Jpn. J. Appl. Phys..

[25] Hirayama T. Rational 3-Dimensional Devices. Proceedings of the 3D Architectures for Semiconductor Integration and Packaging Conference (3D ASIP).

[26] Tong Q.-Y., Cha G., Gafiteanu R., Gösele U. (1994). Low temperature wafer direct bonding. J. Microelectromech. Syst..

[27] Fukushima T., Iwata E., Konno T., Bea J.C., Lee K.W., Tanaka T., Koyanagi M. (2010). Surface tension-driven chip self-assembly with load-free hydrogen fluoride-assisted direct bonding at room temperature for three-dimensional integrated circuits. Appl. Phys. Lett..

[28] Fukushima T., Hashiguchi H., Bea J., Ohara Y., Murugesan M., Lee K.-W., Tanaka T., Koyanagi M. New Chip-to-Wafer 3D Integration Technology Using Hybrid Self-Assembly and Electrostatic Temporary Bonding. Proceedings of the IEEE International Electron Devices Meeting (IEDM).

[29] Hashiguchi H., Yonekura H., Fukushima T., Murugesan M., Kino H., Lee K.-W., Tanaka T., Koyanagi M. Plasma Assisted Multichip-to-Wafer Direct Bonding Technology for Self-Assembly Based 3D Integration. Proceedings of the IEEE 65th Electronic Components and Technology Conference (ECTC).

[30] Stengle R., Tan T., Gösele U. (1989). A model for the silicon wafer bonding process. Jpn. J. Appl. Phys..

[31] Plach T., Hingerl K., Tollabimazraehno S., Hesser G., Dragoi V., Wimplinger M. (2013). Mechanisms for room temperature direct wafer bonding. J. Appl. Phys..

[32] Vandroux L., Cioccio L.D., Gueguen P. (2013). Treatment, Before the Bonding of a Mixed Cu-Oxide Surface, by a Plasma Containing Nitrogen and Hydrogen. U.S. Patent.

[33] Rauer C., Moriceau H., Fournel F., Charvet A.M., Morales C., Rochat N., Vandroux L., Rieutord F., McCormick T., Raduc I. (2013). Treatments of deposited SiOx surfaces enabling low temperature direct bonding. ECS J. Solid State Sci. Technol..

[34] Fournel F., Martin-Cocher C., Radisson D., Larrey V., Beche E., Morales C., Delean P.A., Rieutord F., Moriceaua H. (2015). Water stress corrosion in bonded structures. ECS J. Solid State Sci. Technol..

[35] Maszara W.P., Goetz G., Caviglia A., McKitterick J.B. (1988). Bonding of silicon wafers for silicon-on-insulator. J. Appl. Phys..

[36] Gilman J.J. (1960). Direct measurements of the surface energies of crystals. J. Appl. Phys..

[37] Vallin O., Jonsson K., Lindberg U. (2005). Adhesion quantification methods for wafer bonding. Mater. Sci. Eng..

[38] Sabbione C., di Cioccio L., Vandroux L., Nieto J.-P., Rieutord F. (2012). Low temperature direct bonding mechanisms of tetraethyl orthosilicate based silicon oxide films deposited by plasma enhanced chemical vapor deposition. J. Appl. Phys..

[39] Takagi H., Maeda R., Chung T.R., Hosoda N., Suga T. (1998). Effect of surface roughness on room-temperature wafer bonding by Ar beam surface activation. Jpn. J. Appl. Phys..

[40] Sato K., Ito K., Hata S., Shimokohbe A. (2003). Self-alignment of microparts using liquid surface tension—behavior of micropart and alignment characteristics. Precis. Eng..

[41] Sariola V., Jääskeläinen M., Zhou Q. (2010). Hybrid microassembly combining robotics and water droplet self-alignment. IEEE Trans. Robot..

[42] Arutinov G., Mastrangeli M., Smits E.C.P., van Heck G., den Toonder J.M.J., Dietzel A. (2015). Foil-to-foil system integration through capillary self-alignment directed by laser patterning. J. Microelectromech. Syst..

[43] Ito Y., Fukushima T., Kino H., Lee K.-W., Tanaka T., Koyanagi M. (2016). Impact of chip-edge structures on alignment accuracies of self-assembled dies for microelectronic system integration. J. Microelectromech. Syst..

[44] Ventosa C., Rieutord F., Libralesso L., Morales C., Fournel F., Moriceau H. (2008). Hydrophilic low-temperature direct wafer bonding. J. Appl. Phys..

[45] Ventosa C., Morales C., Libralesso L., Fournel F., Papon A.M., Lafond D., Moriceau H., Penot J.D., Rieutord F. (2009). Mechanism of thermal silicon oxide direct wafer bonding. Electrochem. Solid-State Lett..

[46] Jimbo T., Sakai S., Katuyama K., Ito M., Tomioka H. Thermal Desorption Behavior of Adsorbed Materials on Wafer Surfaces. Proceedings of the IEEE International Symposium on Semiconductor Manufacturing Conference Proceedings.

